# Early Detection Methods for Autism Spectrum Disorder: From Clinical Screening to Multimodal AI

**DOI:** 10.3390/diagnostics16152376

**Published:** 2026-07-28

**Authors:** Wenhao Luo, Zhiwu Yin, Jianbiao Dai

**Affiliations:** 1 Institute for Data Engineering and Science, University of Saint Joseph, Estrada Marginal da Ilha Verde, 14–17, Macao, China; luo.wenhao@usj.edu.mo; 2 Netclass Technology Inc., Shanghai 200436, China; yinzhiwu@netclasstech.com

**Keywords:** autism spectrum disorder, early detection, scoping review, multimodal artificial intelligence, digital phenotyping, explainable AI

## Abstract

Early detection of autism spectrum disorder (ASD) in young children is essential for timely referral, developmental monitoring, and access to early intervention. However, conventional screening and diagnostic pathways often depend on parent-report instruments, episodic clinical observation, and specialist-administered assessments, which may delay identification during the first years of life. This scoping review maps the methodological landscape of early ASD detection from traditional clinical screening to multimodal artificial intelligence (AI). A structured literature search was conducted across major biomedical, psychological, and engineering databases for studies published between January 2010 and May 2026. After screening and eligibility assessment, 65 evidence sources were included in the qualitative synthesis, with additional methodological guidelines used to support reporting and appraisal. The reviewed evidence shows that early ASD detection is increasingly shifting from single-session clinical assessment toward multidimensional risk characterization. Clinical and behavioral screening tools remain the foundation of early identification, while eye tracking, video-based motor analysis, acoustic and vocal biomarkers, electroencephalography (EEG), functional near-infrared spectroscopy (fNIRS), and molecular or genomic indicators provide complementary information across different developmental windows. AI-based methods, including machine learning, deep learning, Transformer architectures, multimodal fusion strategies, and foundation-model-based representation learning, may improve the objective quantification of gaze, movement, vocalization, neural activity, and biological risk. Nevertheless, most AI-assisted systems remain limited by small and heterogeneous datasets, insufficient external validation, population bias, privacy concerns, computational burden, and limited interpretability. This review argues that future early ASD detection systems should be developed as clinician-supervised decision-support tools rather than autonomous diagnostic instruments. Clinically meaningful progress will require robust external validation, privacy-preserving deployment, age-appropriate risk stratification, and intrinsically interpretable architectures that align model outputs with developmental and clinical knowledge.

## 1. Introduction

Autism Spectrum Disorder (ASD) is a highly heterogeneous neurodevelopmental disorder, with core phenotypes manifesting as social communication deficits and restricted, repetitive patterns of behavior [1]. Epidemiological data shows a continuous upward trend in the global prevalence of childhood autism, which brings a heavy public health burden to families and society. Extensive developmental neuroscience and longitudinal tracking studies consistently indicate that the first three years of life (0–36 months) represent the golden window for brain neuroplasticity development [2,3]. Early and intensive behavioral interventions during this period may support more favorable developmental trajectories in language, cognition, and social adaptation [2].

However, the current clinical screening and diagnostic systems suffer from severe structural lags. Traditional screening primarily relies on parent-report questionnaires (e.g., Modified Checklist for Autism in Toddlers (M-CHAT)) alongside episodic manual observations and scale assessments (e.g., Autism Diagnostic Observation Schedule, Second Edition (ADOS-2)) conducted by clinical expert teams within specific medical institutions [4]. This paradigm is not only constrained by a severe shortage of general practitioners and psychiatrists—leading to months-long waitlists—but is also highly prone to diagnostic biases due to the stress reactions of young infants in unfamiliar medical environments [5,6]. Furthermore, the autism symptoms of young children are often intertwined with normal developmental variations, and their behavioral deficits (such as gaze aversion and delayed response to name) are difficult to precisely quantify without continuous monitoring [3,7,8]. Therefore, there is an urgent need within the clinical medical community to develop objective, potentially scalable, and non-invasive automated ultra-early screening technologies.

In recent years, breakthroughs in artificial intelligence (AI) and ubiquitous computing have driven the evolution of autism screening from highly controlled, expensive hardware-dependent laboratory environments to comprehensive digital phenotyping in natural daily living scenarios [9,10]. This shift is increasingly supported by the integration of heterogeneous sensor networks that enable objective, longitudinal tracking of behavioral markers across diverse, non-clinical settings [5]. Through portable sensing terminals such as smartphones, embedded cameras, and miniature microphones, researchers can unobtrusively and non-invasively continuously collect raw video and audio data of young children during natural play and home interactions [9,11]. Deep learning algorithms can extract fine-grained visual and acoustic features from multimodal behavioral data, thereby transforming ambiguous clinical observations into computable and quantifiable digital biomarkers [5,9]. By identifying subtle, sub-clinical patterns—such as micro-saccadic eye movements or high-frequency vocal spectral variance that elude human perception—these models provide an objective substrate for early assessment [12].

Although a growing number of reviews have examined AI-assisted autism detection, important gaps remain in relation to early-childhood populations. For example, a recent systematic review investigated explainable artificial intelligence (XAI) methods in autism assessment but focused primarily on neuroimaging modalities, including functional magnetic resonance imaging (fMRI) and electroencephalography (EEG), across broad age groups. Consequently, the cross-modal integration of heterogeneous audio, video, behavioral, neurophysiological, and biological signals in young children has not been comprehensively synthesized [13]. To address this gap, the present review examines unimodal digital phenotyping approaches [9,14], multimodal fusion strategies [15], and the interpretability and clinical applicability of AI-assisted methods [13,16]. Importantly, it distinguishes approaches that have been directly evaluated in ASD-related cohorts from emerging computational architectures that currently represent methodological opportunities rather than clinically validated screening systems. The review therefore aims to map the available evidence, assess its methodological and translational maturity, and identify priorities for future clinician-supervised research without assuming that emerging AI architectures are ready for routine clinical implementation. Accordingly, this scoping review was guided by the following review questions:

(1) What clinical screening instruments, behavioral observation paradigms, digital phenotyping approaches, neurophysiological markers, molecular or genomic biomarkers, and AI-assisted methods have been investigated for early ASD detection in infants, toddlers, and preschool-aged children?
(2) How are these approaches positioned across different developmental windows, detection targets, data-acquisition contexts, and levels of clinical translational readiness?
(3) What methodological, interpretability-related, ethical, and implementation challenges currently limit the translation of multimodal AI-assisted ASD detection systems into clinician-supervised early screening and risk-stratification pathways?

## 2. Methods

### 2.1. Review Design

This review was designed as a scoping review to map the methodological landscape of early detection methods for autism spectrum disorder (ASD) in young children. The scope of the review included conventional clinical screening instruments, behavioral observation paradigms, eye tracking, video-based motor analysis, acoustic and vocal biomarkers, neurophysiological and neuroimaging markers, molecular and genomic biomarkers, and artificial intelligence-based multimodal detection systems. Scoping review methodology was selected because this review aimed to summarize and categorize a heterogeneous body of evidence rather than estimate a single pooled diagnostic effect size [17,18]. The review was conducted in accordance with the methodological guidance of the Joanna Briggs Institute for scoping reviews and reported according to the Preferred Reporting Items for Systematic Reviews and Meta-Analyses extension for Scoping Reviews (PRISMA-ScR) [17,18]. Given the substantial heterogeneity in study populations, age ranges, detection modalities, reference standards, model architectures, and outcome metrics, a quantitative meta-analysis was not performed.

### 2.2. Search Strategy

A structured literature search was conducted in PubMed/MEDLINE, Scopus, Web of Science Core Collection, IEEE Xplore, and APA PsycINFO. These databases were selected to provide complementary coverage of biomedical and clinical research, psychology and child development, multidisciplinary literature, and artificial intelligence and engineering studies relevant to early autism spectrum disorder (ASD) detection. The search covered publications from 1 January 2010 to 31 May 2026. Google Scholar was additionally used for supplementary forward citation tracking, and the reference lists of relevant empirical studies and review articles were manually screened to identify potentially eligible sources. No formal language restriction was applied at the database-search stage; however, sufficient English-language bibliographic information or an English abstract was required to assess eligibility and extract the relevant methodological information.

The search strategy was structured around four major conceptual domains: (1) autism spectrum disorder; (2) infants, toddlers, preschool-aged children, and early-childhood populations; (3) screening, detection, diagnosis, early identification, risk stratification, or prediction; and (4) clinical, behavioral, digital, neurophysiological, molecular, or artificial intelligence-based detection modalities. Search terms therefore included controlled vocabulary, where available, and free-text terms related to ASD, infants and young children, screening and early detection, clinical screening instruments, behavioral observation, the Modified Checklist for Autism in Toddlers (M-CHAT), the Autism Diagnostic Observation Schedule (ADOS), eye tracking, visual attention, computer vision, pose and movement analysis, acoustic and vocal biomarkers, electroencephalography (EEG), functional near-infrared spectroscopy (fNIRS), neuroimaging, polygenic risk, digital phenotyping, machine learning, deep learning, multimodal fusion, and artificial intelligence.

Database-specific syntax, controlled vocabulary, field tags, and search functions were adapted to the indexing structure of each database. Medical Subject Headings (MeSH) and title/abstract fields were used in PubMed/MEDLINE; title, abstract, and keyword fields were searched in Scopus; Topic searching was used in Web of Science Core Collection; and corresponding keyword and bibliographic search fields were used in IEEE Xplore and APA PsycINFO. To improve the transparency and reproducibility of the review during revision, the database-specific search strategies were reconstructed from the predefined review concepts and rerun on 21 July 2026. Complete database-specific search strategies, database platforms, search fields, coverage periods, search dates, and retrieved record counts are provided in Supplementary Table S1 and Supplementary S1.

### 2.3. Eligibility Criteria

Studies were included if they met the following criteria: (1) focused on ASD screening, early detection, risk stratification, diagnostic support, or early phenotypic characterization; (2) involved infants, toddlers, preschool children, or developmental cohorts relevant to early ASD detection; (3) reported at least one clinical, behavioral, digital, neurophysiological, molecular, or computational detection method; and (4) provided methodological, diagnostic, predictive, or translational information relevant to early ASD detection. Studies were excluded if they focused exclusively on adult populations, did not report ASD-related detection or screening outcomes, were limited to intervention efficacy without detection relevance, lacked sufficient methodological detail, or were purely opinion-based articles. Primary empirical studies constituted the principal evidence base for the appraisal of diagnostic, predictive, and translational validity. Review articles and enabling methodological studies were used for citation tracking, conceptual synthesis, and contextual discussion and were explicitly distinguished from primary empirical evidence during data extraction and narrative synthesis.

### 2.4. Study Selection and Data Extraction

All retrieved records were imported into Zotero for reference management. Duplicate records were identified using DOI, PMID, title, author, and publication-year information and were manually checked before title and abstract screening. Following duplicate removal, 1900 unique records were screened by title and abstract against the predefined eligibility criteria. The first author and reviewer (W.L.) conducted the initial title and abstract screening and full-text eligibility assessment. To reduce the risk of reviewer-dependent selection bias, the second author and reviewer (Z.Y.) independently checked a randomly selected a 20% sample of the title and abstract records (*n* = 380) and a randomly selected a 20% sample of the full-text reports (*n* = 34). The second reviewer completed the independent assessment without reference to the initial screening decisions. Any disagreements regarding study eligibility were discussed by W.L. and Z.Y. and resolved by consensus with reference to the predefined inclusion and exclusion criteria.

Data were extracted by W.L. using a standardized data-extraction form. The extracted information included author and year, country or region, study design, sample size, participant age range, diagnostic or screening reference standard, detection modality, data-acquisition setting, biomarkers or features, computational or statistical method, validation strategy, reported performance metrics, clinical applicability, and major limitations. Z.Y. independently verified the extracted information for a randomly selected 20% sample of the evidence sources included in the qualitative synthesis (*n* = 13). Any discrepancies were resolved through joint re-examination of the original publications and discussion between the two reviewers.

Detection methods were grouped into eight categories: clinical screening and diagnostic instruments; behavioral observation and parent-report tools; eye-tracking and visual-attention markers; video-based motor and pose analysis; acoustic and vocal biomarkers; neurophysiological and neuroimaging biomarkers; molecular and genomic biomarkers; and multimodal or artificial intelligence-based integrated detection systems.

**Figure 1:**
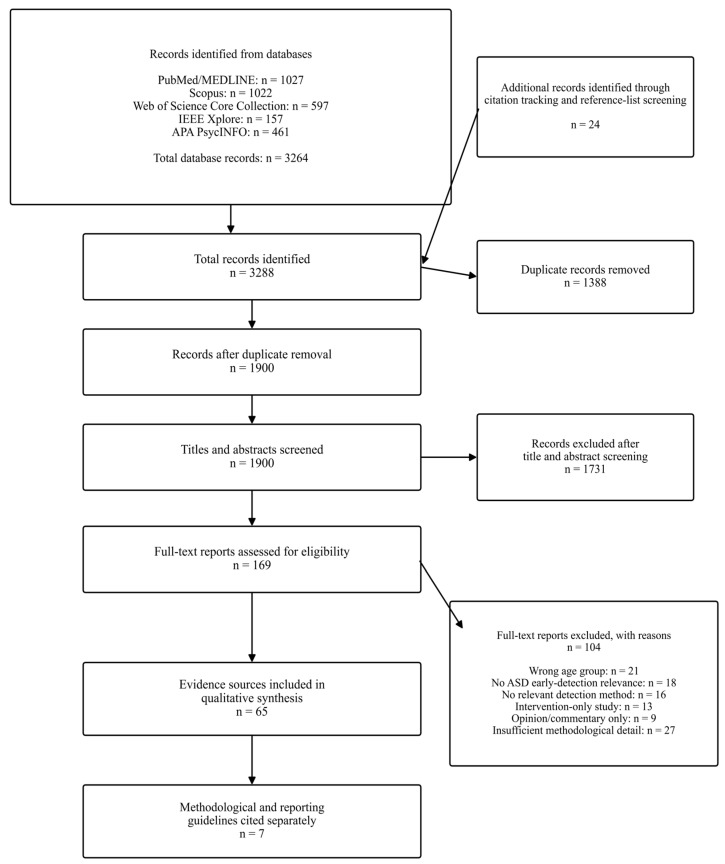
PRISMA-ScR flow diagram of study identification, screening, eligibility assessment, and inclusion.

The study-identification and selection process is summarized in Figure 1. A total of 3264 records were identified from PubMed/MEDLINE, Scopus, Web of Science Core Collection, IEEE Xplore, and APA PsycINFO, and 24 additional records were identified through citation tracking and reference-list screening, resulting in 3288 records. After 1388 duplicate records were removed, 1900 unique records underwent title and abstract screening, of which 1731 were excluded. A total of 169 full-text reports were assessed for eligibility, and 104 were excluded because of an inappropriate age range (*n* = 21), absence of relevance to ASD early detection (*n* = 18), absence of an eligible detection method (*n* = 16), an intervention-only design (*n* = 13), an opinion or commentary format (*n* = 9), or insufficient methodological detail (*n* = 27). Finally, 65 evidence sources were included in the qualitative synthesis. Seven methodological and reporting guideline references were cited separately to support the review design, reporting, and quality-appraisal framework and were not counted as included evidence sources in the PRISMA-ScR selection process.

### 2.5. Evidence Synthesis

The included literature was synthesized narratively and organized according to detection modality, developmental applicability, and clinical translational readiness. For each category, we summarized the core detection target, typical age range, data acquisition burden, representative technologies, main biomarkers, reported diagnostic or predictive performance, and major limitations. Particular attention was paid to the distinction between population-level screening, risk stratification, diagnostic assistance, and definitive clinical diagnosis. Multimodal approaches were further analyzed according to their fusion strategy, including early fusion, late fusion, intermediate fusion, cross-modal attention, graph-based fusion, and foundation-model-based architectures.

### 2.6. Quality and Translational Appraisal

Because the included literature covered diverse methodologies ranging from clinical scales to AI-based multimodal systems, a formal pooled risk-of-bias analysis was not conducted. Studies reporting diagnostic or screening accuracy were appraised with reference to Standards for Reporting Diagnostic Accuracy Studies 2015 (STARD 2015) reporting guideline [19] and Quality Assessment of Diagnostic Accuracy Studies-2 (QUADAS-2) assessment tool [20], with attention to participant selection, index test definition, reference standard, timing, flow, and applicability to early ASD detection [19,20]. For AI-based prediction studies, model reporting and translational quality were considered with reference to transparent reporting of a multivariable prediction model for individual prognosis or diagnosis with artificial intelligence (TRIPOD + AI) reporting guideline [21] and the prediction model risk-of-bias assessment tool with artificial intelligence (PROBAST + AI) assessment tool [22], including sample representativeness, predictor definition, outcome definition, missing-data handling, validation strategy, overfitting risk, calibration, discrimination, transparency, and clinical applicability [21,22]. For studies describing early-stage clinical evaluation of AI decision-support systems, clinical workflow integration, human–AI interaction, safety considerations, and implementation feasibility were additionally considered with reference to Developmental and Exploratory Clinical Investigation of Decision-support systems driven by Artificial Intelligence (DECIDE-AI) reporting guideline [23]. These frameworks were used as interpretive guides to structure the narrative and category-level appraisal; they were not applied as complete study-level checklists, formal domain-based risk-of-bias ratings, or numerical scoring instruments. Accordingly, the appraisal focused on recurrent methodological patterns across detection categories rather than assigning formal ratings to individual studies. Given the substantial heterogeneity in study populations, detection modalities, outcome definitions, and validation strategies, methodological quality was summarized qualitatively by detection category rather than converted into a single numerical score. Table 1 provides a structured category-level overview of the dominant study designs, validation profiles, evidence maturity, and recurrent methodological concerns across the major early ASD detection approaches.

**Table 1:** Qualitative summary of methodological quality and evidence maturity among primary empirical studies.

Detection Approach	Typical Evidence Base	Validation Profile	Evidence Maturity	Main Methodological Concerns and Interpretation
Clinical and behavioral screening	Prospective or cross-sectional studies using standardized screening or clinical assessment instruments	Repeated clinical use, although performance varies across populations and settings	More established	Parental-report bias, participant selection, variable reference standards, and dependence on trained clinicians; suitable for clinical screening but not sufficient as a standalone diagnostic pathway
Eye-tracking and visual attention	Case-control and prospective studies comparing gaze-related markers with clinical assessment	Some independent clinical comparisons, but limited multisite and population-diverse validation	Emerging	Small or selected samples, calibration failure, hardware variability, and stimulus dependence; best interpreted as an adjunctive risk-stratification measure
Video-based motor and pose analysis	Mainly retrospective, case-control, or proof-of-concept studies	Predominantly cross-validation or internal hold-out testing	Preliminary	Small datasets, possible participant-level data leakage, annotation bias, environmental variability, and overfitting; external validation remains necessary
Acoustic and vocal biomarkers	Retrospective analyses of crying, vocalization, prosody, or caregiver–child interaction	Mainly internal validation	Preliminary	Language and dialect bias, background noise, class imbalance, limited reproducibility, and small samples; findings may not generalize across linguistic populations
Neurophysiological biomarkers	Small case-control or cohort studies using EEG, fNIRS, or related neural signals	Mostly internal validation with limited independent replication	Preliminary	Motion artifacts, protocol heterogeneity, equipment burden, small samples, and limited external validation; currently more suitable for biological risk characterization than population screening
Molecular and genomic biomarkers	Cohort, secondary-data, or genetic-association studies	Limited ASD-specific validation for early detection and limited ancestry diversity	Preliminary	Ancestry bias, indirect association with behavioral diagnosis, uncertain calibration, ethical concerns, and limited clinical interpretability
Multimodal AI approaches	Retrospective multimodal studies and early proof-of-concept systems	Mostly internal validation; prospective clinical and workflow evaluation remains uncommon	Exploratory	Overfitting, dataset shift, missing calibration, limited interpretability, lack of external validation, and insufficient evidence of clinical workflow integration

Note: STARD 2015, QUADAS-2, TRIPOD+AL PROBAST+AI and DECIDE-AI informed the appraisal domains but were not applied as formal study-level scoring instruments.

## 3. Early ASD Detection Methods: From Clinical Screening to Biological Biomarkers

### 3.1. Clinical and Behavioral Screening Methods

Conventional clinical and behavioral screening methods remain the foundation of early ASD identification. Parent-report instruments, such as the Modified Checklist for Autism in Toddlers (M-CHAT), provide accessible tools for population-level screening, whereas structured clinical observation and standardized diagnostic assessments help clinicians evaluate social communication deficits and restricted or repetitive behaviors [24]. In early development, behavioral paradigms such as response to name, joint attention, social smiling, atypical social engagement, and early non-social behaviors provide clinically meaningful indicators of ASD risk [2,7,8]. However, these approaches are constrained by parental recall bias, short observation windows, delayed symptom manifestation, and dependence on trained professionals. Therefore, traditional clinical screening should be viewed as the entry point for early identification.

### 3.2. Visual Attention and Eye Tracking

Atypical social visual engagement is one of the most extensively studied early behavioral markers associated with ASD. During typical development, attention to faces, eyes, biological motion, and caregiver–child interaction support the emergence of joint attention and later social communication. Eye-tracking studies have shown that young children with ASD may display altered scan paths, reduced attention to socially relevant stimuli, and atypical visual preferences during social and non-social viewing paradigms [25,26]. Preferential-looking studies further suggest that some children with ASD show increased attention to repetitive geometric motion compared with social stimuli [27].

Recent studies have strengthened the clinical relevance of eye tracking by testing objective measures of social visual engagement against expert clinical diagnosis and by developing shorter, lower-burden paradigms suitable for young children [6,28,29,30]. Nevertheless, eye tracking is not a standalone diagnostic tool. Its performance can be affected by calibration quality, child compliance, stimulus design, hardware variability, and testing environment. Therefore, eye tracking is best positioned as an objective adjunct for early risk stratification rather than a replacement for clinical diagnosis.

### 3.3. Kinematics and Motor Patterns

Motor atypicalities and restricted or repetitive behaviors are important behavioral dimensions in ASD assessment. In early childhood, atypical motor patterns may include unusual spontaneous movements, body rocking, repetitive hand or finger movements, and altered whole-body coordination. Longitudinal evidence also suggests that atypical spatiotemporal patterns of spontaneous movement in early life may be associated with later ASD risk [31].

Video-based motion analysis has enabled more objective quantification of these behaviors. Computer vision methods, including 2D pose estimation and skeleton-based modeling, can extract body keypoints and derive kinematic features such as movement trajectory, velocity, acceleration, and postural coordination [32,33]. Broader advances in skeleton-based action recognition provide methodological support for modeling temporal movement patterns from video data [34]. These approaches may reduce the burden of manual video coding and support scalable behavioral assessment, but their clinical reliability remains sensitive to camera angle, occlusion, lighting, background complexity, annotation quality, and dataset representativeness [35].

Current evidence suggests that video-based motor analysis is promising for objective behavioral phenotyping, particularly when distinguishing ASD-related motor profiles from other neurodevelopmental conditions or when embedded into structured interaction settings such as robot-mediated activities [36,37]. However, these methods should be framed as complementary tools for behavioral quantification rather than definitive diagnostic instruments.

### 3.4. Acoustic and Vocal Biomarkers

Acoustic and vocal biomarkers provide another low-burden route for capturing early developmental differences associated with ASD. Before the emergence of fluent speech, infant crying, pre-linguistic vocalizations, atypical prosody, and caregiver–child turn-taking patterns may contain information relevant to social communication development [14,38]. Automated acoustic analysis can extract features such as fundamental frequency, pitch variability, spectral structure, vocalization rate, response latency, and conversational timing.

Early computational studies used machine learning models, including support vector machines and recurrent neural networks, to analyze natural vocalizations in young children and explore their potential for earlier ASD identification [39]. In interactive contexts, acoustic-prosodic and turn-taking features may further reflect atypical timing and reciprocity during caregiver–child communication [40]. Recent corpus-building work has also expanded the field toward cross-linguistic assessment, including annotated speech resources for autistic children in non-English contexts [41,42].

Despite these advantages, acoustic biomarkers are affected by language, dialect, recording quality, background noise, microphone placement, and interaction context. Therefore, speech and vocal analysis should be interpreted as complementary developmental signals rather than independent diagnostic evidence.

### 3.5. Neurophysiological Biomarkers: Electroencephalography (EEG)

Neurophysiological and neuroimaging markers extend early ASD detection from observable behavior to underlying neural processes. EEG is particularly relevant because of its high temporal resolution and its ability to capture neural synchrony, signal complexity, and functional connectivity during early development. Prior work has suggested that EEG complexity and related neural features may be associated with ASD risk, although their stability and clinical utility require further validation in larger longitudinal cohorts [43].

Recent AI-based EEG studies have applied convolutional networks, graph neural networks, and Transformer-based architectures to model multichannel brain signals and functional connectivity patterns [44,45]. In addition, systematic reviews have highlighted the emerging use of EEG and fNIRS with machine learning or deep-learning methods for early ASD-related classification and risk assessment [46]. Related neurophysiological approaches, including magnetoencephalography (MEG)-based fusion models, also suggest the potential value of brain-signal-based machine learning for ASD risk characterization [47].

These approaches may provide mechanistic insight and complementary biological information, but they remain limited by small samples, motion artifacts, protocol variability, equipment burden, and limited external validation. Therefore, neurophysiological markers should be interpreted as potential tools for biological risk stratification rather than first-line population screening methods.

### 3.6. Molecular and Genomic Biomarkers: Polygenic Risk Scores and Foundation Models

Molecular and genomic biomarkers provide information on biological susceptibility rather than direct behavioral diagnosis. ASD has a substantial genetic contribution, and genetic risk may involve rare variants, copy number variations, de novo mutations, common polygenic variation, and broader regulatory mechanisms. In this context, AI-based genomic models may help characterize complex variant effects and nonlinear biological interactions, particularly in high-dimensional genomic and regulatory data [48].

Polygenic risk scores and related machine learning approaches have been explored for psychiatric risk prediction and subgroup stratification, but their clinical application to early ASD detection remains preliminary and requires cautious interpretation, especially across diverse ancestral populations [49,50]. Beyond genomics alone, multi-omics machine learning frameworks may integrate genomic, transcriptomic, and other biological data to support mechanistic understanding and biological stratification [51]. Recent foundation-model approaches in network biology further suggest that transfer learning may help extract context-aware representations from large-scale biological data, although their direct utility for ASD early screening has not yet been established [52].

Emerging gene–brain–behavior frameworks provide a promising direction for linking biological susceptibility, neural development, and observable behavioral phenotypes [53,54]. Overall, molecular and genomic biomarkers may contribute to baseline risk estimation and biological subtype discovery, but they should not be positioned as standalone screening or diagnostic tools. Their clinical translation is constrained by population heterogeneity, ethical concerns, interpretability, cost, and the need to connect biological risk markers with observable developmental outcomes.

Across the reviewed modalities, several recurring methodological patterns were evident. Established clinical instruments benefited from repeated use, clearer reference standards, and greater interpretability, but remained dependent on trained personnel and episodic observation. Eye-tracking, acoustic, video-based, and neurophysiological approaches offered more objective and potentially repeatable measurements, yet were commonly limited by small or selected samples, protocol and equipment heterogeneity, internal validation, and uncertain calibration. Molecular and genomic approaches provided early biological-risk information but showed limited ASD-specific validation, ancestry diversity, and direct linkage to clinically observable outcomes. Multimodal AI could integrate complementary signals, but its apparent gains were often difficult to separate from dataset composition, information leakage, or model complexity. Overall, increasing technological sophistication was not consistently accompanied by stronger external or clinical validation.

**Figure 2:**
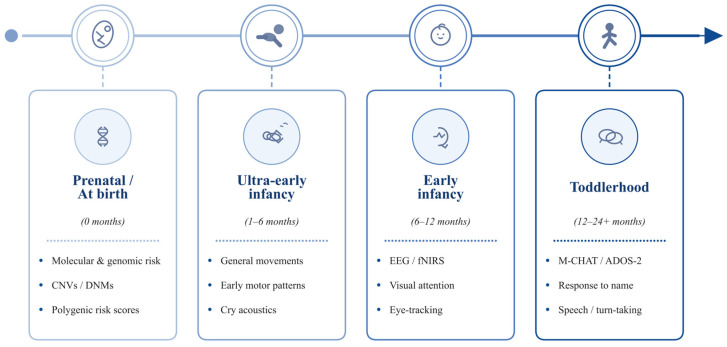
Conceptual developmental timeline of early ASD detection modalities. Note: The developmental stages represent an author-derived synthesis of the reviewed literature rather than evidence from a single longitudinal study or a fixed clinical pathway. The indicated age ranges should therefore be interpreted as approximate developmental windows reported across heterogeneous studies.

To further clarify the developmental applicability of different early ASD detection approaches, Figure 2 provides a conceptual timeline of detection modalities across prenatal, infant, and toddler developmental stages. Molecular and genomic markers may provide early biological susceptibility information from birth or even prenatally, whereas general movements, early motor patterns, and cry acoustics may offer low-burden behavioral signals during the first months of life [31,38]. From approximately 6 to 12 months, neurophysiological measures and visual attention paradigms, including EEG, fNIRS, and eye tracking, may capture emerging neural and social-attentional atypicalities [25,43,46]. During toddlerhood, parent-report screening, structured behavioral observation, response-to-name paradigms, and speech or turn-taking analysis become more clinically interpretable and developmentally observable [2,7,24]. This timeline emphasizes that early ASD detection should be understood as a staged and complementary framework rather than a single-modality diagnostic pathway, as shown in Figure 2. 

In summary, early ASD detection methods should be understood as complementary rather than competing approaches. Clinical tools provide accessibility and interpretability, digital behavioral phenotyping improves objective quantification of early social, motor, and vocal signals, and neurobiological or molecular markers may support biological risk stratification. However, none of these methods currently provides sufficient evidence to replace expert clinical diagnosis. The most clinically realistic direction is therefore to integrate these approaches into a staged risk-stratification framework, in which different modalities contribute age-appropriate information for earlier referral, longitudinal monitoring, and clinician-supervised decision-making.

## 4. Multimodal AI and Emerging Algorithmic Architectures for Early ASD Detection

For clarity, Section 3 summarizes clinical, behavioral, neurophysiological, and biological approaches that have been primarily evaluated in ASD-related populations. Section 4 focuses on emerging computational architectures whose relevance ranges from ASD-specific proof-of-concept evidence to transferable methods developed outside ASD research. Their inclusion is intended to map methodological possibilities and should not be interpreted as equivalent evidence of clinical readiness.

### 4.1. From Traditional Machine Learning to Spatiotemporal Transformers

In the early stages of digital phenotyping for autism, algorithmic architectures primarily relied on a “two-stage pipeline”: first extracting discrete statistics of the face, eyes, and voice through hand-crafted features or shallow convolutional neural networks (CNNs), and then feeding them into a support vector machine (SVM), random forest (RF), or traditional autoencoder for feature dimensionality reduction and classification [33,55]. Although these methods have relatively low computational requirements, their ability to model complex long-term dependencies in heterogeneous and lengthy home-video recordings is limited, potentially increasing the risk of false-positive classifications.

More recent studies have explored Transformer-based architectures and real-time detection models because of their capacity to represent long-range temporal dependencies and complex spatial patterns in video data. These properties are potentially relevant to naturalistic ASD research, in which behavioral recordings may be lengthy, noisy, and developmentally dynamic. However, their clinical relevance to early ASD screening remains uncertain.

General computer vision and human–action–recognition research has also introduced architectures such as Video Swin Transformers and Video Swin-CLSTM models, which combine spatial feature extraction with temporal modeling [56,57,58]. Similarly, real-time object-detection frameworks may provide technical components for identifying or localizing behavioral events in video streams [59]. These developments demonstrate transferable computational capabilities, but most were not originally designed or clinically validated for early ASD detection. Their inclusion in this review therefore reflects their potential methodological relevance rather than evidence of established screening effectiveness.

Within ASD-specific research, these architectures may support future analyses of temporally structured behaviors, such as gaze shifts, repetitive movements, social orientation, or response-to-name events. These potential applications remain exploratory in the context of early ASD detection. Table 2 summarizes the progression from directly evaluated ASD-related approaches to emerging algorithmic directions, while distinguishing established evidence from methods that remain primarily exploratory.

### 4.2. Foundation Models and Multimodal Representation Learning

Foundation models and multimodal representation learning may provide new methodological opportunities for early ASD detection, particularly in contexts where annotated clinical datasets are limited. General-purpose visual models such as Segment Anything [64] and DINOv2 [65] may support automated segmentation, feature extraction, and representation learning from complex home videos, while biomedical vision-language models such as LLaVA-Med [63] suggest the potential value of aligning visual representations with clinical text. Unified multimodal representation frameworks, such as OmniBind and UniBind, further demonstrate how heterogeneous data types may be mapped into shared embedding spaces [62,66].

However, these models should not be regarded as clinically validated tools for early ASD screening. In naturalistic home videos, large models may capture background characteristics, camera quality, caregiver behavior, or demographic cues rather than clinically meaningful developmental phenotypes. Accordingly, foundation models should currently be viewed as methodological enablers for future ASD research rather than established clinical screening systems.

### 4.3. Data-Level, Decision-Level, and Attention-Level Fusion Mechanisms

The phenotypes of ASD in early life are often covert and dynamically intertwined. Relying solely on unimodal signals (such as only looking at eye movements or only listening to voice) for classification often hits a generalization bottleneck due to the one-sidedness of the feature dimensions. Therefore, designing robust fusion strategies for heterogeneous data represents a major methodological challenge in current research and development:

Data-level Fusion (Early Fusion): Directly concatenating the feature vectors of audio and video during the feature extraction stage [5]. The flaw in this method is that when facing high-dimensional continuous video and one-dimensional temporal audio signals, it is highly prone to dimensional imbalance, causing the model to overly bias toward a dominant modality.

Decision-level Fusion (Late Fusion): In decision-level fusion, visual, auditory, postural, and clinical-scale models are trained and evaluated independently, and their outputs are subsequently integrated to generate a final risk estimate through weighted averaging, majority voting, logistic regression, or other ensemble strategies [47]. This strategy has practical advantages because each modality-specific model can be optimized according to the characteristics of its own data stream. For example, visual models may focus on gaze allocation, facial orientation, or repetitive motor behavior, whereas acoustic models may emphasize vocal timing, prosodic variation, or response latency. By keeping these modality-specific pipelines separate, late fusion can reduce the direct technical conflict caused by heterogeneous data formats, sampling rates, feature dimensions, and missing modalities. It may also improve operational flexibility in real-world screening contexts, where some data sources may be unavailable or unreliable.

Recent methodological research has increasingly explored dynamic alignment networks based on cross-modal attention. In principle, these architectures may help model temporally aligned relationships between heterogeneous signals. For example, in a response-to-name paradigm, an audio representation of the caregiver’s verbal cue could be aligned with subsequent video-based estimates of head orientation, gaze direction, or body movement. Such alignment may support the characterization of temporally coordinated behavioral patterns while retaining modality-specific information. However, their added clinical value for early ASD risk stratification remains uncertain. As shown in Table 3, unimodal detection modalities for early ASD screening mainly include behavioral observation, eye tracking, vocal/acoustic analysis, neurophysiological signals, motor-pattern analysis, and molecular or genetic biomarkers. Building on unimodal detection approaches, Table 4 presents the main multimodal fusion strategies that combine complementary information across different data modalities for early ASD detection.

To further evaluate the clinical practicability of different detection modalities, operational burden and active assessment time should be considered alongside diagnostic or predictive performance. Standardized clinician-administered assessments such as the ADOS-2 commonly require approximately 40–60 min of structured, one-to-one observation by trained professionals, although the total clinical pathway may vary across settings. Dedicated laboratory-based systems, including fixed eye-tracking devices, may require additional time for calibration, participant preparation, and task administration. By comparison, smartphone- or camera-based digital phenotyping approaches may reduce the duration of active data collection when brief behavioral tasks are embedded within naturalistic home or play settings. However, shorter acquisition time should not be interpreted as evidence of equivalent clinical validity. The practical value of these approaches depends on data quality, child engagement, calibration requirements, automated quality control, and validation in representative clinical populations.

Cross-scale fusion represents a further but still preliminary research direction. In principle, genetic risk indicators, neurophysiological connectivity measures, and video–audio behavioral phenotypes could be integrated to examine relationships among biological susceptibility, neural development, and observable behavior [53,54]. Interpretable architectures such as GAMI-Net and multimodal hypernetwork frameworks illustrate possible computational strategies for modeling heterogeneous data while retaining a degree of transparency [16,70]. However, these methods should not be regarded as validated ASD screening systems unless they have been directly evaluated in relevant early-childhood cohorts. Gene–brain–behavior fusion therefore remains an exploratory framework that requires ASD-specific validation, external replication, calibration assessment, and evaluation of clinical utility.

**Figure 3:**
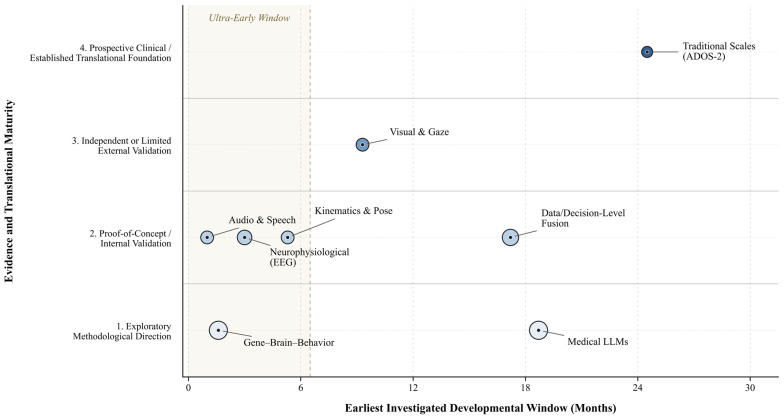
Conceptual mapping of developmental applicability and evidence maturity across early ASD detection approaches.

Building on the modality-specific and cross-scale evidence summarized above, Figure 3 presents a qualitative conceptual mapping of major early ASD detection approaches according to three dimensions: the earliest developmental window in which each approach has been investigated, its current level of evidence and translational maturity, and its relative methodological complexity. Because the included studies reported heterogeneous outcome measures, including accuracy, AUROC, sensitivity, specificity, and F1 score, these metrics were not combined into a common quantitative performance axis. Instead, evidence maturity was categorized according to study design, validation strategy, independence of the evaluation cohort, and the extent of prospective or workflow-based clinical assessment.

This mapping highlights an important translational tension: approaches that are clinically established and readily interpretable may be applied later in development, whereas some ultra-early biological, neurophysiological, and computational modalities may provide earlier risk-related signals but remain supported primarily by proof-of-concept or internally validated evidence. Accordingly, the development of AI-assisted ASD screening should be evaluated not only in terms of discrimination performance, but also according to robustness, calibration, external validity, interpretability, operational feasibility, and suitability for clinician-supervised decision support. As shown in Figure 3, earlier developmental applicability does not necessarily imply greater clinical or translational maturity.

## 5. Methodological Limitations, Generalizability, and Deployment Constraints

### 5.1. Data Scarcity, Generalization Challenges, and Edge Deployment Constraints

Although AI-assisted approaches have reported promising classification results in controlled research settings, the methodological characteristics of the current evidence substantially limit the interpretation and clinical transferability of these findings. Many studies rely on relatively small samples, retrospective analyses, single-center cohorts, or case–control designs comparing children with established ASD diagnoses against neurotypical controls. Such designs are useful for early proof-of-concept development but may overestimate performance because they do not fully represent the diagnostically heterogeneous populations encountered in routine developmental assessment. In clinical settings, children referred for ASD evaluation may also present with language delay, attention-deficit/hyperactivity disorder, intellectual disability, anxiety, motor impairment, or other developmental conditions. Models trained on highly separated case–control groups may therefore learn differences that are easier to detect than those required for clinically meaningful differential risk stratification.

Overfitting and information leakage represent additional concerns, particularly in studies using high-dimensional video, audio, eye-tracking, EEG, or multimodal data. When multiple frames, clips, vocal segments, or signal windows from the same participant are treated as independent observations, random data splitting may place information from one child in both the training and test sets. This participant-level leakage can produce overly optimistic estimates of model performance. Similar inflation may occur when feature selection, data augmentation, normalization, or hyperparameter optimization is conducted before the separation of training and test data. Future studies should therefore use participant-level data partitioning, nested validation where appropriate, prespecified analytical pipelines, and fully independent test cohorts.

Internal cross-validation alone does not establish that a model will generalize across clinical sites or populations. Differences in recruitment procedures, ASD prevalence, diagnostic criteria, age distribution, language, culture, socioeconomic context, recording equipment, stimulus design, and data-acquisition protocols may produce substantial dataset shift. A model developed using carefully controlled recordings from a single research center may perform differently when applied to home videos, primary-care settings, community screening programs, or populations with different demographic and linguistic characteristics. External validation should therefore include geographically, culturally, and clinically distinct cohorts, with subgroup analyses used to evaluate whether performance remains stable across sex, age, language, ethnicity, developmental level, and co-occurring conditions.

The current literature also places greater emphasis on discrimination metrics than on calibration and clinical utility. Accuracy, sensitivity, specificity, F1 score, and the area under the receiver operating characteristic curve describe different aspects of performance, but they do not indicate whether predicted risk probabilities correspond to observed clinical outcomes. Poorly calibrated models may assign excessively high or low risk estimates even when overall discrimination appears acceptable. This is particularly important in population-level screening, where disease prevalence and referral thresholds directly influence positive predictive value, false-positive burden, parental anxiety, and demand for specialist services. Future studies should report calibration plots or quantitative calibration measures, confidence intervals, prespecified decision thresholds, and clinically relevant predictive values in addition to conventional discrimination metrics.

Reproducibility remains another important limitation. Differences in preprocessing, participant exclusion, missing-data handling, model selection, class-imbalance correction, and performance reporting make it difficult to compare results across studies. In many cases, insufficient reporting of model architecture, training procedures, software implementation, random-seed control, or data availability prevents independent replication. More transparent reporting in accordance with TRIPOD + AI, together with participant-level validation, accessible analytical code where ethically permissible, and standardized reporting of demographic and clinical characteristics, would strengthen the reliability of future evidence.

These methodological limitations are compounded by practical deployment constraints. Multimodal models may require substantial computational resources and may experience latency or power-consumption challenges when implemented in primary-care clinics, community settings, or home-based edge devices. At the same time, video, audio, and physiological recordings of young children contain highly sensitive personal and family information. Privacy-preserving approaches such as on-device processing, secure data governance, and federated learning may reduce some risks, but their effects on model performance, system reliability, and equity require empirical evaluation. Consequently, current AI-assisted ASD detection models should be interpreted primarily as research or decision-support tools until their robustness, calibration, reproducibility, and clinical benefit have been demonstrated through prospective, multicenter, and externally validated studies.

Despite their potential, AI-assisted approaches are not yet ready for routine implementation in pediatric screening pathways. Clinical translation will require prospective multicenter validation in geographically, demographically, and clinically diverse populations, together with clearly defined intended use and appropriate regulatory evaluation. Implementation studies should examine not only predictive performance but also calibration, failed or incomplete recordings, clinician overrides, referral outcomes, workflow burden, and compatibility with existing pediatric and developmental assessment services. Adoption will also depend on staff training, clinician acceptance, interoperability, cost-effectiveness, and locally applicable reimbursement arrangements. In addition, responsibilities for false-negative or false-positive outputs, data security, model updates, and final clinical decisions will need to be clarified among developers, healthcare institutions, and supervising clinicians. Because these regulatory, economic, and medico-legal conditions vary across jurisdictions, current AI-assisted methods should be regarded as clinician-supervised decision-support tools rather than autonomous screening or diagnostic systems.

### 5.2. From Black-Box Post-Hoc Attribution to Intrinsic Interpretability

A major barrier preventing automated ASD detection technologies from entering real clinical workflows lies in the limited interpretability of modern neural network models. In pediatric developmental assessment, clinicians are unlikely to rely solely on an algorithmic risk score, particularly when the output may influence parental concern, referral decisions, and access to early intervention. For example, a system that simply reports that “the risk of ASD is 94%” provides limited clinical value unless it can explain which developmental, behavioral, or biological signals contributed to that prediction. In this context, explainability is not merely a technical supplement but a key requirement for clinical accountability, professional trust, and responsible deployment in early-childhood screening [71,72].

Current research has increasingly attempted to integrate post-hoc explainability tools such as SHapley Additive exPlanations (SHAP), Local Interpretable Model-agnostic Explanations (LIME), saliency maps, gradient-weighted class activation mapping (Grad-CAM), and gradient-based attribution methods into AI-assisted ASD detection systems. These methods can provide useful information about model behavior by identifying which features, regions, or signal segments contribute most strongly to a prediction [13,71,72]. For instance, in video-based detection, saliency maps may highlight a child’s face, hands, body posture, or repetitive motor movements; in acoustic analysis, attribution methods may identify atypical vocal intensity, high-frequency crying segments, or abnormal prosodic patterns; and in EEG-based models, feature attribution may indicate specific electrodes, frequency bands, or connectivity features associated with elevated risk. These tools are valuable for preliminary model inspection and may help researchers identify potential confounding variables or failure cases.

However, this review suggests caution regarding an excessive reliance on post-hoc explanation methods. Post-hoc tools usually approximate the behavior of a trained black-box model after the prediction has already been made. Therefore, they do not necessarily reveal whether the model has learned clinically meaningful developmental mechanisms or merely captured dataset-specific artifacts. This concern is particularly relevant in early ASD detection because behavioral and physiological signals are often temporally structured, context-dependent, and multimodal. For example, a hand-flapping movement may be clinically meaningful only when interpreted together with gaze direction, social context, vocal response, and developmental age. Similarly, reduced response to name, atypical gaze allocation, delayed vocal turn-taking, and repetitive motor behavior should be understood as coordinated developmental patterns rather than isolated features. Perturbation-based post-hoc explanations may fail to preserve these temporal and contextual dependencies, producing explanations that appear visually plausible but remain clinically incomplete or potentially misleading [13,71].

To reduce concerns regarding black-box decision-making, future ASD detection systems should move beyond the conventional “black-box model plus post-hoc explanation” paradigm and place greater emphasis on intrinsically interpretable architectures. Such models should embed clinically meaningful reasoning structures into the design of the algorithm itself rather than merely explaining the output afterward. Concept Bottleneck Models provide a useful example of this direction, because they require the model to first map raw multimodal inputs into human-readable intermediate concepts before generating a final risk estimate [69]. In early ASD screening, these intermediate concepts could include reduced eye contact, atypical gaze shifting, limited social smiling, delayed response to name, repetitive hand movements, abnormal vocal turn-taking, atypical prosody, EEG connectivity abnormalities, or molecular risk indicators. By introducing such a concept layer, the model’s prediction becomes more closely aligned with clinical developmental knowledge.

Under this architecture, a multimodal AI system may still retain the representational power of deep learning for high-dimensional video, audio, eye-tracking, EEG, or genomic inputs, but its decision pathway becomes more clinically reviewable. Instead of directly transforming raw behavioral signals into a single risk probability, the system first estimates interpretable developmental concepts and then uses these concepts to support the final classification or risk-stratification decision. For example, the model may indicate that an elevated risk estimate is associated with reduced social orienting, atypical gaze allocation, delayed vocal reciprocity, and repetitive motor patterns, rather than providing an unexplained probability score. This structure allows clinicians to evaluate whether the model’s reasoning is consistent with established clinical knowledge and to review, question, or override algorithmic outputs when necessary.

Interpretable hypernetworks and structured multimodal models may further support this transition by representing heterogeneous clinical, behavioral, and biological features while preserving some degree of transparency [16]. This is particularly important for cross-scale ASD detection, where video-audio behavioral phenotypes may be integrated with neurophysiological signals and genetic risk indicators. Without interpretable design, such fusion systems may increase predictive complexity while reducing clinical transparency. Therefore, next-generation models should clarify not only the final risk estimate but also the relative contribution of each modality and the developmental meaning of the extracted features.

Intrinsically interpretable architectures could provide a more clinically reviewable decision pathway by linking model outputs to candidate developmental concepts that clinicians can examine. In principle, this structure may make model uncertainty and reasoning more visible and could support future evaluation of clinician trust and shared decision-making. However, these potential benefits have not yet been established in prospective ASD screening workflows. Future studies should therefore evaluate whether interpretable models improve clinical understanding, decision consistency, referral quality, and family communication compared with conventional black-box models. As an author-proposed conceptual framework arising from this review, this layered logic may be provisionally termed the Ethical Hierarchy of Explainability (EHE). It is intended to organize considerations relating to technical transparency, clinical alignment, and ethical actionability and should not be interpreted as an empirically validated clinical or technical framework. At the highest level, the EHE should support actionable and ethical interpretability. Architectures such as Concept Bottleneck Models (CBMs) may be used to encourage the AI system to express its intermediate reasoning in human-readable biological or behavioral concepts before generating a final risk estimate. If a multi-omics model flags a high risk for ASD, it should ideally indicate whether the risk is mainly associated with a de novo mutation, atypical EEG alpha-band asymmetry, abnormal gaze trajectory, or a combination of these factors. Such a layered approach may help reduce the risk of algorithmic determinism in pediatric populations and support clinicians in planning appropriate follow-up, referral, and early intervention strategies [72].

### 5.3. Balancing Developmental Timing and Predictive Robustness: Toward Clinician-Supervised Early Risk Stratification

A persistent challenge in early ASD screening is the balance between developmental timing and predictive robustness. Traditional clinical assessments and structured behavioral observations are relatively interpretable and clinically grounded, but they often become most informative when social-communication behaviors are sufficiently observable, usually during toddlerhood. By contrast, ultra-early signals such as cry acoustics, spontaneous motor patterns, neurophysiological markers, and genomic risk indicators may shift risk detection toward earlier developmental windows, but their standalone predictive reliability remains variable and usually requires further validation in larger and more diverse cohorts [2,31,38,43]. Therefore, the core challenge is not simply to identify the earliest possible biomarker, but to determine how early signals can be integrated, validated, and interpreted in a clinically meaningful way.

The age-accuracy trade-off also reflects the heterogeneity of ASD itself. Early behavioral atypicalities may be subtle, intermittent, and highly dependent on developmental stage, family environment, and recording context. A single modality may capture only one dimension of risk: eye tracking may reflect social attention, acoustic analysis may capture vocal development, pose estimation may quantify motor patterns, EEG may reflect neural connectivity, and genomic indicators may suggest biological susceptibility. However, none of these modalities alone can fully represent the developmental complexity of ASD. This is why multimodal and cross-scale approaches are increasingly important. By integrating behavioral phenotypes, neurophysiological signals, and biological risk indicators, future systems may improve early risk stratification while reducing overreliance on any single marker [25,28,53,54].

Nevertheless, such systems should be understood as decision-support tools rather than autonomous diagnostic instruments. In clinical practice, the purpose of early AI-assisted screening should be to identify children who may benefit from further developmental evaluation, longitudinal monitoring, or early intervention referral, not to replace expert diagnosis. Future research should therefore evaluate ASD detection systems using a broader translational framework that includes not only reported accuracy or area under the receiver operating characteristic curve (AUC/AUROC), but also age of applicability, external validation, calibration, false-positive burden, privacy protection, interpretability, family acceptability, and clinician usability. A clinically useful system should help reduce referral delays and support age-appropriate risk stratification while remaining transparent, ethically governed, and supervised by qualified professionals.

Taken together, balancing developmental timing and predictive robustness does not imply pursuing the earliest possible prediction without adequate clinical validation. Rather, it requires a staged screening framework in which different modalities contribute age-appropriate evidence across development. Biological and neurophysiological markers may support early risk awareness, digital behavioral phenotyping may provide objective monitoring of emerging social, vocal, and motor patterns, and clinical assessment remains essential for diagnostic confirmation and intervention planning. In this sense, the future of AI-assisted ASD detection lies in integrating early signals into a robust, interpretable, and clinician-supervised pathway for developmental risk stratification.

## 6. Conclusions

Early detection of autism spectrum disorder represents a clinically significant and rapidly evolving field in child developmental health. As highlighted throughout this review, early ASD detection is no longer limited to episodic, clinician-dependent assessment, but is increasingly shaped by the integration of clinical screening, behavioral observation, digital phenotyping, neurophysiological markers, molecular biomarkers, and multimodal artificial intelligence. Rather than functioning as isolated diagnostic signals, these modalities reflect complementary dimensions of early developmental risk. Clinical tools provide accessibility and interpretability, digital behavioral phenotyping enables objective quantification of social, motor, and vocal patterns, and biological markers may contribute to risk stratification and mechanistic understanding. Together, these approaches indicate that early ASD detection should be understood as a multidimensional and staged framework rather than a single-modality diagnostic pathway.

However, methodological progress alone is insufficient for clinical translation. Current AI-assisted ASD detection systems remain constrained by small and heterogeneous datasets, limited external validation, population bias, privacy concerns, computational burden, and insufficient interpretability. Future systems should therefore be developed as clinician-supervised decision-support tools rather than autonomous diagnostic instruments. Clinically meaningful progress will require age-appropriate risk stratification, robust external validation, privacy-preserving deployment, family acceptability, and interpretable architectures that align model outputs with developmental and clinical knowledge. Routine implementation will additionally require prospective multicenter validation, regulatory and workflow evaluation, evidence of economic value, clinician acceptance, and clearly defined medico-legal accountability. Technical performance alone should therefore not be interpreted as sufficient evidence of clinical readiness. In this sense, the future of AI-assisted ASD screening lies not in replacing expert diagnosis, but in building robust, transparent, and ethically governed systems that can support earlier referral, longitudinal monitoring, and individualized developmental care.

## Figures and Tables

**Table 2:** Established and emerging AI architectures relevant to early ASD detection.

Evolutionary Stage	Core Paradigm	Key Literature	Key Technological Highlights
Traditional Assessment Era (Pre-2015)	Clinical Scale Observation	[24]	Standardized clinical scales; established the gold standard for behavioral diagnosis.
Unimodal Computation Era (2015–2020)	Feature Engineering and Shallow Machine Learning	[11,32]	Utilized preliminary pose estimation and eye-tracking features to achieve unimodal behavioral quantification.
Deep Fusion Exploration Era (2020–2023)	Deep Learning and Data/Decision- Level Fusion	[4,60]	Introduction of multi-layer neural networks, attempting to mine complex atypical interaction features from videos and audio.
Deep Alignment Architecture Era (2023–2025)	Cross-Modal Attention Mechanisms	[15,61]	Cross-attention provides a potential mechanism for modeling spatiotemporal relationships across heterogeneous modalities; however, its added predictive and clinical value for early ASD detection remains insufficiently validated.
Universal Foundation Model Era (2025–Future)	Intrinsic Interpretability and Foundation Models	[62,63,64]	Foundation models may support unified multimodal representation and clinical-text alignment, but their clinical utility for early ASD detection has not yet been established.

**Table 3:** Unimodal detection modalities for early ASD detection.

Modality/Strategy	Detection Target	Representative Architecture	Key References
Unimodal: Vision and Eye Tracking	Decreased social visual engagement, gaze aversion, atypical preference for geometric repetitive movements.	CNN, SVM, Vision Transformer (ViT)	[12,28,29]
Unimodal: Kinematics and Pose	Stereotypic movements (e.g., hand-flapping, body rocking), atypical general movements (GMs), gait abnormalities.	2D/3D skeleton extraction (OpenPose), Spatiotemporal LSTM (CLSTM), Video Swin	[32,35,67]
Unimodal: Audio and Speech	Cry spectral variations (fundamental frequency distortion), atypical pre-linguistic vocalizations, delayed turn-taking in conversational interactions.	1D-CNN, Bi-LSTM, Self-supervised acoustic Foundation Models (HuBERT)	[14,38,40]
Unimodal: Neurophysiological (EEG and fNIRS)	Atypical cortical connectivity, resting-state Gamma-band hyper-connectivity, Alpha-band asymmetry	1D-CNN, Spatial–Temporal Graph Convolutional Network (ST-GCN), EEG-Conformer	[44,46]
Unimodal: Molecular and Genomic	De Novo Mutations (DNMs), Copy Number Variations (CNVs), Polygenic Risk Scores (PRS) for biological subgroup stratification.	Deep CNNs (e.g., DeepSEA), Transformer-based Genomic Foundation Models (Geneformer)	[49,50]

Note: copy number variations = CNVs, de novo mutations = DNMs, and polygenic risk scores = PRSs, long short-term memory = LSTM, convolutional long short-term memory = CLSTM, one-dimensional convolutional neural network = 1D-CNN, bidirectional long short-term memory = Bi-LSTM, Hidden-Unit BERT = HuBERT.

**Table 4:** Multimodal fusion strategies for early ASD detection.

Modality/Strategy	Detection Target (Digital Biomarkers)	Representative Architecture	Key References
Multimodal: Data/Decision-Level Fusion (Early/Late Fusion)	Simple concatenation or result voting of visual, audio, and clinical scale data to output a comprehensive risk score.	Feature Concatenation + Random Forest (RF) or Multilayer Perceptron (MLP)	[5,47,60]
Multimodal: Spatiotemporal Cross-Attention Fusion	Potential modeling of temporally coordinated relationships across heterogeneous behavioral or physiological signals.	Spatiotemporal Graph Convolutional Network (ST-GCN)	[15,67]
Multimodal: Cross-Scale Fusion (Gene–Brain–Behavior)	Alignment of innate genetic susceptibility (Prior), neuro-topological anomalies (Intermediate), and behavioral deficits (Endpoint).	Hypernetworks, Generative Machine Learning	[54]
Frontier: Multimodal Large Models (Foundation Models and LLMs)	Potential support for unified cross-modal representation and clinician-readable summaries; direct evidence for ASD-specific zero-shot reasoning or clinically reliable explanation generation remains limited.	Visual instruction-tuned large models (LLaVA-Med), omni-scenario unified binding spaces (Omnibind)	[62,63,66,68,69]

Note: LLMs = large language models.

## Data Availability

No new data were created or analyzed in this study. Data sharing is not applicable to this article.

## Supplementary Materials

The following supporting information can be downloaded at: https://www.mdpi.com/article/10.3390/diagnostics16152376/s1, Table S1: Database-specific search characteristics. S1: Complete Database-Specific Search Strategies.
