# Supplementary material for: Early Detection Methods for Autism Spectrum Disorder: From Clinical Screening to Multimodal AI
Source: Diagnostics (Basel). 2026 Jul 28;16(15):2376. doi: 10.3390/diagnostics16152376 (PMC13464867; doi:10.3390/diagnostics16152376)
Supplement: Supplementary file 1 [file diagnostics-16-02376-s001.zip › diagnostics-4441394-supplementary.pdf]

Supplementary Materials

Table S1. Database-specific search characteristics

Database	Platform	Search field	Search date	Coverage period	Records retrieved
PubMed/MEDLINE	PubMed	MeSH and Title/Abstract	21 July 2026	1 January 2010–31 May 2026	1,027
Scopus	Elsevier	Article Title, Abstract, Keywords	21 July 2026	January 2010–May 2026	1,022
Web of Science Core Collection	Clarivate	Topic	21 July 2026	January 2010–May 2026	597
IEEE Xplore	IEEE	Document/metadata search	21 July 2026	January 2010–May 2026	157
APA PsycINFO	EBSCOhost	Keyword/bibliographic fields	21 July 2026	January 2010–May 2026	461

S1. Complete Database-Specific Search Strategies

S1.1 PubMed/MEDLINE

("Autism Spectrum Disorder"[Mesh] OR autis*[Title/Abstract] OR ASD[Title/Abstract]) AND (infant*[Title/Abstract] OR toddler*[Title/Abstract] OR preschool*[Title/Abstract] OR "young child"[Title/Abstract] OR "young children"[Title/Abstract] OR "early childhood"[Title/Abstract]) AND ("early detection"[Title/Abstract] OR "early screening"[Title/Abstract] OR "early diagnosis"[Title/Abstract] OR "early identification"[Title/Abstract] OR "risk stratification"[Title/Abstract] OR "diagnostic support"[Title/Abstract] OR screening[Title/Abstract]) AND ("screening tool"[Title/Abstract] OR "clinical scale"[Title/Abstract] OR "behavioral observation"[Title/Abstract] OR "M-CHAT"[Title/Abstract] OR ADOS[Title/Abstract] OR "eye tracking"[Title/Abstract] OR "eye-tracking"[Title/Abstract] OR "visual attention"[Title/Abstract] OR "computer vision"[Title/Abstract] OR "pose estimation"[Title/Abstract] OR "motor pattern"[Title/Abstract] OR "motor patterns"[Title/Abstract] OR kinematic*[Title/Abstract] OR acoustic*[Title/Abstract] OR vocal*[Title/Abstract] OR "speech analysis"[Title/Abstract] OR EEG[Title/Abstract] OR electroencephalograph*[Title/Abstract] OR fNIRS[Title/Abstract] OR "functional near-infrared spectroscopy"[Title/Abstract] OR neuroimaging[Title/Abstract] OR "polygenic risk score"[Title/Abstract] OR biomarker*[Title/Abstract] OR "digital phenotype"[Title/Abstract] OR "digital phenotyping"[Title/Abstract] OR "machine learning"[Title/Abstract] OR "deep

learning"[Title/Abstract] OR "artificial intelligence"[Title/Abstract] OR multimodal[Title/Abstract] OR "multi-modal"[Title/Abstract] OR "cross-modal"[Title/Abstract]) AND ("2010/01/01"[Date - Publication] : "2026/05/31"[Date - Publication])

S1.2 Scopus

TITLE-ABS-KEY (("autism spectrum disorder" OR autism OR ASD) AND (infant* OR toddler* OR preschool* OR "young child" OR "young children" OR "early childhood") AND ("early detection" OR "early screening" OR "early diagnosis" OR "early identification" OR "risk stratification" OR "diagnostic support") AND ("screening tool" OR "clinical scale" OR "behavioral observation" OR "M-CHAT" OR ADOS OR "eye tracking" OR "visual attention" OR "computer vision" OR "pose estimation" OR "motor pattern" OR "motor patterns" OR kinematic* OR acoustic* OR vocal* OR "speech analysis" OR EEG OR electroencephalograph* OR fNIRS OR "functional near-infrared spectroscopy" OR neuroimaging OR "polygenic risk score" OR biomarker* OR "digital phenotype" OR "digital phenotyping" OR "machine learning" OR "deep learning" OR "artificial intelligence" OR multimodal OR "multi-modal" OR "cross-modal")) AND PUBYEAR > 2009 AND PUBYEAR < 2027

S1.3 Web of Science Core Collection

```
TS=(
  ("autism spectrum disorder" OR autism OR ASD)
  AND
  (infant* OR toddler* OR preschool* OR "young child" OR
   "young children" OR "early childhood")
  AND
  ("early detection" OR "early screening" OR "early diagnosis" OR
   "early identification" OR "risk stratification" OR
   "diagnostic support")
  AND
  ("screening tool" OR "clinical scale" OR "behavioral observation" OR
   "M-CHAT" OR ADOS OR "eye tracking" OR "eye-tracking" OR
   "visual attention" OR "computer vision" OR "pose estimation" OR
   "motor pattern" OR "motor patterns" OR kinematic* OR acoustic* OR
   vocal* OR "speech analysis" OR EEG OR electroencephalograph* OR
   fNIRS OR "functional near-infrared spectroscopy" OR neuroimaging OR
   "polygenic risk score" OR biomarker* OR "digital phenotype" OR
   "digital phenotyping" OR "machine learning" OR "deep learning" OR
   "artificial intelligence" OR multimodal OR "multi-modal" OR
   "cross-modal")
)
AND PY=(2010-2026)
```

S1.4 IEEE Xplore

("autism spectrum disorder" OR autism OR ASD) AND (infant OR toddler OR preschool OR "young child" OR "young children" OR "early childhood") AND ("early detection" OR "early screening" OR "early diagnosis" OR "early identification" OR "risk stratification" OR "diagnostic support") AND ("screening tool" OR "clinical scale" OR "behavioral observation" OR "M-CHAT" OR ADOS OR "eye tracking" OR "visual attention" OR "computer vision" OR "pose estimation" OR "motor pattern" OR kinematic OR acoustic OR vocal OR "speech analysis" OR EEG OR electroencephalography OR fNIRS OR "functional near-infrared spectroscopy" OR neuroimaging OR "polygenic risk score" OR biomarker OR "digital phenotype" OR "digital phenotyping" OR "machine learning" OR "deep learning" OR "artificial intelligence" OR multimodal OR "multi-modal" OR "cross-modal")

S1.5 APA PsycINFO

("autism spectrum disorder" OR autism OR ASD)
AND
(infant* OR toddler* OR preschool* OR "young child" OR "young children" OR "early childhood")
AND
("early detection" OR "early screening" OR "early diagnosis" OR "early identification" OR "risk stratification" OR "diagnostic support")
AND
(
"screening tool"
OR "clinical scale"
OR "behavioral observation"
OR "M-CHAT"
OR ADOS
OR "eye tracking"
OR "visual attention"
OR "computer vision"
OR "pose estimation"
OR "motor pattern"
OR kinematic*
OR acoustic*
OR vocal*
OR "speech analysis"
OR EEG
OR electroencephalograph*
OR fNIRS
OR "functional near-infrared spectroscopy"
OR neuroimaging
OR "polygenic risk score"
OR biomarker*
OR "digital phenotype"
OR "digital phenotyping"

OR "machine learning"
OR "deep learning"
OR "artificial intelligence"
OR multimodal
OR "multi-modal"
OR "cross-modal"
)
